# 

**Published:** 1941

**Authors:** 


### The Medical Library of the University of Bristol

WITH WHICH IS INCORPORATED

### The Library of the Bristol Medico-Chirurgical Society

*The following donations have been received since the publication of the list in May,* 1941.

November, 1941.

| | |
|---|---|
| Army Medical Library, Washington (1) .. .. | 1 volume. |
| British Dental Association (2) .. .. .. .. | 1 volume. |
| Brompton Hospital (3) .. .. .. .. .. | 1 volume. |
| Mrs. Collingwood (4) .. .. .. .. .. | 5 volumes. |
| Dr. O. C. M. Davies (5) .. .. .. .. | 1 volume. |
| Dr. I. Fischer (6) .. .. .. .. .. | 2 volumes. |
| Professor E. W. Hey Groves (7) .. .. .. | 25 volumes. |
| Hendry-Connell Research Foundation (8) .. .. | 1 volume. |
| T. W. Lloyd (9) .. .. .. .. .. .. | 1 volume. |
| Michell Clarke Fund (10) .. .. .. .. | 4 volumes. |
| Shaw Bequest (11) .. .. .. .. .. | 2 volumes. |
| C. H. Walker, Esq. (12) .. .. .. .. | 65 volumes. |

Unbound periodicals have also been received from Dr. S. B. Adams, Dr. R. F. Barbour, Professor E. W. Hey Groves, Dr. T. Milling, Professor C. Bruce Perry, Dr. J. Odery Symes, Dr. C. H. Walker.

### THE ONE HUNDRED AND SEVENTY-FOURTH LIST OF BOOKS.

The figures in round brackets refer to the figures after the names of the donors. The books to which no such figures have been attached have either been bought from the Library Fund or received through the *Journal*.

**Amor, A. J.** .. .. .. *An X-ray Atlas of Silicosis* .. .. .. .. .. .. 1941

**Anderson, W.** .. .. *The Early Treatment of War Wounds* .. .. 1941

**Ballance, Sir C.** .. .. *Lister Memorial Lecture*, 1933 .. .. .. (7) 1933

**Bardenheuer, B.** .. .. *Allgemeine Lehre von den Frakturen und Luxationen* .. .. .. .. .. .. .. .. (7) 1907

**Bauer, B. A.** .. .. *Woman* .. .. .. .. .. .. .. .. .. .. (4) 1927

**Beaumont, G. E., and Dodds, E. C.** *Recent Advances in Medicine* 10th Ed. 1941

**Berry, R. J. A., and Gordon, R. G.** *The Mental Defective* .. .. .. (7) 1931

**Best, C. H., and Taylor, N. B.** *The Physiological Basis of Medical Practice* 2nd Ed. (10) 1939

**Bonnin, J. G.** .. .. *A Complete Outline of Fractures* .. .. .. .. .. 1941

**Burke, E. T.** .. .. .. *Venereal Diseases* .. .. .. .. .. .. .. .. .. 1940

**Burrows, H.** .. .. .. *Pitfalls of Surgery* .. .. .. 2nd Ed. (7) 1925

**Cameron, J. L.** .. .. *Gynæcological Operations* .. .. .. .. .. .. 1941

**Choulant, L.** .. .. *Geschichte und Bibliographie der Anatomischen Abbildung* .. .. .. .. .. .. .. .. .. .. 1852

**Clarke, A.** .. .. .. *An Essay on Warm, Cold, and Vapour Bathing* 5th Ed. 1820

*Clinical Histories of a Series of Cancer Cases Treated with Ensol only* (8) 1940

*Contributions to Medical and Biological Research dedicated to Sir William Osler in honour of his 70th birthday.* 2 vols. 1919

**Costler, A., and others** *Encyclopædia of Sexual Knowledge* .. .. (4) 1934

**Culpeper, N.** .. .. .. *English Physician and Complete Herbal* .. .. 1938

**Curie, J. R.** .. .. .. *Hygiene* .. .. .. .. .. .. .. .. .. .. (10) 1938

**Davidson, M.** .. .. *A Practical Manual of Diseases of the Chest* 2nd Ed. 1941

**De Kruif, P.** .. .. *The Fight for Life* .. .. .. .. .. .. .. .. 1938

**Ellis, H.** .. .. .. .. *Studies in the Psychology of Sex.* Vols. 1, 3—6 1920

**Fischer, I.** .. .. .. *Biographisches Lexicon.* 2 Bde. .. .. .. .. 1933

**Forel, A.** .. .. .. *Die sexuelle Frage.* 4te *Auft* .. .. .. .. 1906

**Galbraith, A. M.** .. .. *Four Epochs of a Woman's Life* .. .. .. (4) 1907

**Glaister, J., and Brash, J. C.** *Medico-legal Aspects of the Ruxton Case* (5) 1937

**Gray, H.** .. .. .. *Anatomy—Descriptive and Surgical* 12th Ed. (4) 1890

**Head, H.** .. .. .. *Aphasia and Kindred Disorders of Speech* (11) 1926

**Hobhouse, R. W.** .. *Life of Christian Samuel Hahnemann—Founder of Homœopathy* .. .. .. .. .. .. .. .. 1933

*Hunterian Orations for* 1921, 1923, 1927, 1928, 1941 .. .. (7) 1921–41

**Hutchison, R., and Hunter, D.** *Clinical Methods* .. .. .. 11th Ed. 1941

*Index-catalogue of Surgeon General's Office* 4th Ser. Vol. V. E—F (1) 1940

**Jones, R., and Ridlon, J.** *Contributions to Orthopædic Surgery* .. .. (7) N.D.

**Jamieson, E. B.** .. .. *Illustrations of Regional Anatomy. Sections* I—V. 3rd Ed. (7) 1941

**Lloyd, T. W.** .. .. *On the Aetiology of Acholuric Family Jaundice* (9) 1940

**McDowall, R. J. S.** .. *Biological Introduction to Psychology* .. .. .. 1941

**Maingot, R.** .. .. .. *Technique of Gastric Operation* .. .. .. .. .. 1941

**Martin, F. B.** .. .. *The Joy of Living.* 2 vols. .. .. .. .. .. (7) 1933

**Mitchiner, P. H., and Cowell, E. M.** *Medical Organization and Surgical Practice in Air Raids* .. .. 2nd Ed. (7) 1941

**Moncrieff, A. A.** ed. .. *Nursing and Diseases of Sick Children* 3rd Ed. 1941

**Norbury, L. E. C.** .. *Carcinoma of the Rectum* .. .. .. .. (7) 1941

**Notter and Firth** .. .. *Hygiene.* 10th ed. rev. by L. C. Adam and E. J. Boome .. .. .. .. .. .. .. (10) 1941

**Quincy, J.** .. .. .. *Compleat English Dispensatory* .. .. 2nd Ed. 1719

**Rogers, L., and Muir, E.** *Leprosy* .. .. .. .. .. .. .. .. 2nd Ed. 1940

**Rolleston, G.**.. .. .. *Harveian Oration,* 1873 .. .. .. .. .. .. .. 1873

**Rolleston, H. D.** (ed.).. *Encyclopædia of Medical Practice—Surveys and Abstracts,* 1940 .. .. .. .. .. .. .. .. 1941

—— *Cumulative Supplement,* 1940 .. .. .. .. 1941

**Rolleston, H. D., and Moncrieff, A. A.** (Eds.). *Diet in Health and Disease* 1939

**Rolleston, H. D., and Moncrieff, A. A.** (Eds.). *Modern Diagnosis* .. .. 1940

**Spenger, W. G.** .. .. *Animal Experiments and Surgery* .. .. (7) 1920

**Stopes, M. C.** .. .. *Radiant Motherhood* .. .. .. .. .. .. (4) 1920

**Streeter, W. A.** .. .. *The New Healing* .. .. .. 2nd Ed. (7) 1932

**Trotter, W.** .. .. .. *Collected Papers of Wilfred Trotter, F.R.S.* .. 1941

**Wall, C.** .. .. .. *The History of the Surgeons' Company,* 1745–1800 1937

**Wallace, J. S.** .. .. *The Teeth and Health* .. .. .. .. .. .. (2) 1926

**Watson-Jones, R.**.. .. *Fractures and Other Bone and Joint Injuries* 2nd Ed. 1941

**Webb-Johnson, A. E.** *The First Hundred Years* .. .. .. .. .. (7) 1935

**Webb-Johnson, A. E.** *Bradshaw Lecture,* 1940 .. .. .. .. .. .. (7) 1940

**Webb-Johnson, A. E.** *Surgical Aspects of Typhoid and Paratyphoid Fevers* .. .. .. .. .. .. .. .. .. (7) 1919

**Weindler, F.** .. .. *Geschichte der gynäkologisch—anatomischen Abbildung* .. .. .. .. .. .. .. .. .. 1908

**Whitman, R.** .. .. *Orthopædic Surgery* .. .. .. .. .. 9th Ed. (7) 1930

### TRANSACTIONS, REPORTS, JOURNALS, ETC.

Brompton Hospital Reports. Vol. IX, 1940 .. .. .. .. .. .. .. (3) 1941

Collected Papers of the Mayo Clinic and Mayo Foundation. XXXII, 1940 (10) 1941

Medical Annual, 1941 .. .. .. .. .. .. .. .. .. .. .. .. .. 1941

Offprints of Articles by Dr. Fischer from German periodicals .. .. (6) —

### Meetings of Societies

### Bristol Medico-Chirurgical Society

### British Medical Association

A meeting of the Bristol Division of the British Medical Association was held in the Reception Room at the University on Thursday, 20th November, at 3 p.m., when Dr. J. A. Nixon gave a British Medical Association lecture on **"The Treatment of Gas Casualties."** Dr. H. M. Golding, D.F.C., was in the Chair.

### Publications Received

From British Pharmacopœdia Commission :

*Fourth Addendum to the British Pharmacopœia*, 1932.

From Messrs. Cassell & Co. Ltd. :

*Berkeley's Handbook of Midwifery.* 11th Edition. Price 8s. 6d.

*Dental Materia Medica, Pharmacology and Therapeutics.* By Dilling and Hallam. Price 13s. 6d.

From Messrs. Lawrence and Wishart, Ltd. :

*Lectures on Conditioned Reflexes.* By Pavlov. Price 8s. 6d.

From Messrs. H. K. Lewis & Co. Ltd. :

*A Textbook on the Nursing and Diseases of Sick Children for Nurses.* By various authors, edited by Alan Moncrieff, M.D., F.R.C.P. Third Edition. Price 21s.

*Common Skin Diseases.* By A. C. Roxburgh. Sixth Edition. Price 16s.

From Messrs. E. and S. Livingstone :

*Fractures vnd Other Bone and Joint Injuries.* By R. Watson-Jones, B.Sc., M.Ch.Orth., F.R.C.S. Second Edition. Price 50s.

From OXFORD UNIVERSITY PRESS :

*The Collected Papers of Wilfred Trotter, F.R.S.* Price 10s. 6d.
*Technique of Gastric Operations.* By RODNEY MAINGOT, F.R.C.S. Price 15s.
*The Early Treatment of War Wounds.* By WILLIAM ANDERSON, O.B.E., M.B., Ch.B., F.R.C.S. Price 5s.
*The Treatment of Burns.* By A. B. WALLACE, M.B., F.R.C.S., M.Sc. Price 5s.

From JOHN WRIGHT & SONS LTD. :

*The Medical Annual,* 1941. Price 23s.
*British Journal of Surgery.* No. 112. April, 1941. No. 113. July, 1941. Price 12s. 6d. each.
*Warwick and Tunstall's First Aid to the Injured and Sick.* Ed. NORMAN HAMMER. Eighteenth Edition. Price 3s. 6d.
*Massage and Remedial Exercises.* By NOEL M. TIDY. Fifth Edition. Price 17s. 6d.

### Local Medical Notes

The C.B. (Military Division) has been conferred on Colonel (Temporary Major-General) P. S. Tomlinson, D.S.O., A.M.S., for distinguished services in the Middle East.

**Hunterian Professors, R.C.S.**

Mr. J. T. Chesterman has been elected to deliver one lecture on "Evaluation and Treatment of Factors involved in Post-lobectomy Collapse of the Lungs."

Mr. F. W. Willway has been elected to deliver one lecture on the "Role of Surgery in Mental Disease."

**University of Aberdeen.**—On 10th July, 1941, the Honorary Degree of LL.D. was conferred (*in absentia*) on Dr. A. W. Falconer, C.B.E., D.S.O., M.D., F.R.C.P., Principal and Vice-Chancellor of the University of Cape Town.

Under the auspices of the Bristol Branch of the Society for Cultural Relations between the peoples of the British Commonwealth and the U.S.S.R., Dr. Benjamin Farrington, Professor of Classics at Swansea University, delivered a lecture on "Medicine from Ancient Greece to Soviet Russia" at the Victoria Rooms, on Wednesday, 16th July. His address appears on page 45 of this issue.

EXAMINATION RESULTS.

**University of Bristol.**—Students of the University have recently passed the following examinations :—

M.B., CH.B.—*First Examination. Pass :* J. H. Beatson, D. E. Bolt, E. R. Duchesne, S. H. Dunce, M. B. Lennard, J. C. Little, H. S. Paull, C. D. R. Pengelly, R. Simpson-White, M. de V. Varian, R. Want, G. Winternitz. *In Chemistry and Physics* :* Kathleen Blott, Jean Tregear.

* Completing the examination.

M.B., CH.B.—*Second Examination. Pass :* J. A. Ardis, R. I. Bodman, C. K. Bridge, Marjorie J. Dix, Margaret P. Drew, Susanna Foss, E. C. Hamlyn[1], Kathleen C. Iles, Flavia Z. L. B. James, P. A. James, Christine M. H. Jones, D. M. M. Jones, M. H. Kerby, G. E. P. Lee, R. Maggs, F. I. Tovey[1 2]. *In Group I only :* J. M. Allen, W. A. Heaton-Ward, Helen M. Holt.

M.B., CH.B.—*Final Examination* (*Section I*). *Pass :* R. C. Blackney, Jocelyn Brodie, Joyce W. Brown, Hester M. Hammond, Mary Jacob, A. H. M. Oakes, A. F. Rogers.

M.B., CH.B.—*Final Examination* (*with First Class Honours*) : G. S. Andrews[3 4], Marie L. Bartlett[3 5]. (*With Second Class Honours*) *:* Cecil Mary Drillien[3], J. C. Valentine[6]. *Pass :* R. C. Blackney, N. J. Brown, J. D. Cardale, G. T. Cribb, J. H. P. Davies-Sage[4], Mary L. Hunt, D. S. Maunsell, H. R. Newman, Christine M. Rendell, F. B. Richards, M. O. Skelton, J. H. Wilkins. *In Group I only :* K. L. Brown, S. J. V. Gilson. *In Group II only :* Joyce W. Brown, Jean A. Butt, J. B. Tucker*.

B.D.S.—*First Examination. Pass :* A. N. R. Gibson.

B.D.S.—*Second Examination. Pass :* I. A. Morgan, Evelyn M. Pitt.

B.D.S.—*Third Examination. Pass :* R. D. Hepburn[7 8], C. R. W. Birkett[7] †, J. R. V. B. Gibson[7] †, Jean A. M. C. Gordon-Ralph[7], J. B. K. Mann[7].

B.D.S.—*Final Examination. Pass : In Section I only :* F. C. Bryant, P. B. Carey, Margaret J. Griffith.

L.D.S.—*First Examination. Pass :* J. F. V. Woolley.

L.D.S.—*Second Examination. Pass :* A. R. Brook, C. Gibbs, L. H. Valentine.

L.D.S.—*Third Examination. Pass :* W. D. Arnold[9] *, D. A. Holmes[7] *, W. E. C. Chaplin[8] *, J. Hornsby[8 9], B. A. Oliver[8 9], R. L. Yendell[8 9], H. B. Doubt[7], Alice B. U. Leigh[7], R. E. May[7], R. H. Shipway[7], R. C. Taylor[7], W. H. E. Turnbull[7], J. B. Westlake[7], F. E. R. Williams[7], J. B. Wood[7], J. W. B. Kay[8], H. Sheffield[9].

L.D.S.—*Final Examination. Pass : Section II:* G. K. G. Jennings*, G. J. Mills*, R. S. Saxton*, R. M. Sharp*. *In Section I :* Jean Bryant.

**Conjoint Board.**

L.R.C.P., M.R.C.S.—*First Examination. Pass : In Physiology :* F. W. P. Clutterbuck.

L.R.C.P., M.R.C.S.—*Final Examination. Pass : In Pathology :* B. C. E. Aldous. *In Medicine :* Margot H. Dickson, G. Steiner. *In Midwifery :* F. Frank, H. Jelinek.

[1] With Distinction in Anatomy.
[2] With Distinction in Physiology.
[3] With Distinction in Surgery.
[4] With Distinction in Obstetrics.
[5] With Distinction in Medicine.
[6] With Distinction in Public Health.
[7] In Dental Anatomy.
[8] In Dental Mechanics and Properties of Materials.
[9] In Dental Materia Medica.
* Completing the examination.
† With Distinction.

APPOINTMENTS.

**Bristol Royal Infirmary.**—Senior Resident Medical Officer : Betty Fox, M.B., B.S., M.R.C.S., L.R.C.P. Resident Anæsthetist : W. Maidlow, M.R.C.S., L.R.C.P., D.A. House Surgeons to the Casualty Department : R. D. R. Shanks, M.B., Ch.B., J. K. Lewis, M.B., Ch.B. House Surgeons to the Fracture Department : Betty Fox, J. D. Cardale, M.B., Ch.B. House Surgeon to Mr. Rendle Short and Mr. Paul : G. S. Andrews, M.B., Ch.B. ; to Mr. Chitty : R. C. Blackney, M.B., Ch.B. ; to Mr. Adams : Cecil Drillien, M.B., Ch.B. ; to Mr. Tasker and Mr. FitzGibbon : D. S. Maunsell, M.B., Ch.B. House Physician to Dr. Carleton and Dr. Perry : Marie Bartlett, M.B., Ch.B. ; to Dr. Herepath and Dr. Sutton : N. J. Brown, M.B., Ch.B. ; to Dr. Birrell and Dr. Orr-Ewing : J. C. Valentine, M.B., Ch.B. ; to Dr. Todd : P. Legat.

**Bristol General Hospital.**—Senior Resident Medical Officer : R. G. Boyd, M.B., Ch.B. House Surgeon to the Casualty Department : C. J. R. Jacob, M.R.C.S., L.R.C.P. House Surgeon to Mr. Moore and Mr. Wood : H. R. Newman, M.B., Ch.B. House Surgeons to the E.N.T. Department : J. H. G. Morris, M.R.C.S., L.R.C.P., L.D.S. ; G. Schwizer, M.R.C.S., L.R.C.P. House Surgeons to the Gynæcological Department : R. G. Boyd, S. B. Richards, M.B., Ch.B. House Surgeon to the Skin and Radium Departments : Mary Hunt, M.B., Ch.B.

**Bristol Royal Hospital, Weston Branch.**—House Physician : Megan Jones, M.B., Ch.B., M.R.C.S., L.R.C.P. House Surgeon : Christine Rendell, M.B., Ch.B.

### Index

Aberdeen, University of.—Honorary Degree, 102

American Ambulance, Great Britain, 27

Appointments, 102

Book Reviews, 22, 91

Boyle, the late Mr. H. Edmond G. (Editorial Note), 95

Bristol Medico-Chirurgical Society.—Clinical Meeting, 101

Bristol University.—
Appointments, 104
Examination Results, 36, 102
Medical Library, 32, 98

British Medical Association.—Bristol Division Meetings (Editorial Note), 95

British Medical Association.—Bristol Division Report, 101

Casualty Surgery of Air Raids, 74

Experiences of First Aid in Air Raids, 57

Farrington, B.—Medicine from Ancient Greece to Soviet Russia, 45

Francis, the late Prof. F. E. (Editorial Note), 30

George Medal awarded to two Bristol Nurses (Editorial Note), 29

Guirdham, A.—Sex Hormones and Blood-Pressure, 19

Hemphill, R. E.—Importance of the First Year of War in Mental Disease, 45

Hunterian Professors, 102

Importance of the First Year of War in Mental Disease.—R. E. Hemphill, 45

Matrons of the General Hospital and Children's Hospital (Editorial Note), 30

Medical Privilege and Responsibility (Editorial Note), 95

Medicine from Ancient Greece to Soviet Russia.—B. Farrington, 45

Obituaries—

H. Elwin Harris, 97

W. H. C. Newnham, 31

Sex Hormones and Blood-Pressure.—A. Guirdham, 19

Short, A. Rendle.—Thyrotoxicosis, 1

Thyrotoxicosis.—A. Rendle Short, 1

Wright, John, & Sons Ltd. (Editorial Note) 30

